# Brain Structural Covariance Network Topology in Remitted Posttraumatic Stress Disorder

**DOI:** 10.3389/fpsyt.2018.00090

**Published:** 2018-03-29

**Authors:** Delin Sun, Sarah L. Davis, Courtney C. Haswell, Chelsea A. Swanson, Mid-Atlantic MIRECC Workgroup, Jean C. Beckham, Kevin S. LaBar, John A. Fairbank, Rajendra A. Morey

**Affiliations:** ^1^Department of Veteran Affairs (VA) Mid-Atlantic Mental Illness Research, Education and Clinical Center, Durham, NC, United States; ^2^Duke-UNC Brain Imaging and Analysis Center, Duke University, Durham, NC, United States; ^3^Department of Psychiatry and Behavioral Sciences, Duke University, Durham, NC, United States

**Keywords:** posttraumatic stress disorder, remission, structural covariance network, cortical thickness, centrality

## Abstract

Posttraumatic stress disorder (PTSD) is a prevalent, chronic disorder with high psychiatric morbidity; however, a substantial portion of affected individuals experience remission after onset. Alterations in brain network topology derived from cortical thickness correlations are associated with PTSD, but the effects of remitted symptoms on network topology remain essentially unexplored. In this cross-sectional study, US military veterans (*N* = 317) were partitioned into three diagnostic groups, current PTSD (CURR-PTSD, *N* = 101), remitted PTSD with lifetime but no current PTSD (REMIT-PTSD, *N* = 35), and trauma-exposed controls (CONTROL, *n* = 181). Cortical thickness was assessed for 148 cortical regions (nodes) and suprathreshold interregional partial correlations across subjects constituted connections (edges) in each group. Four centrality measures were compared with characterize between-group differences. The REMIT-PTSD and CONTROL groups showed greater centrality in left frontal pole than the CURR-PTSD group. The REMIT-PTSD group showed greater centrality in right subcallosal gyrus than the other two groups. Both REMIT-PTSD and CURR-PTSD groups showed greater centrality in right superior frontal sulcus than CONTROL group. The centrality in right subcallosal gyrus, left frontal pole, and right superior frontal sulcus may play a role in remission, current symptoms, and PTSD history, respectively. The network centrality changes in critical brain regions and structural networks are associated with remitted PTSD, which typically coincides with enhanced functional behaviors, better emotion regulation, and improved cognitive processing. These brain regions and associated networks may be candidates for developing novel therapies for PTSD. Longitudinal work is needed to characterize vulnerability to chronic PTSD, and resilience to unremitting PTSD.

## Introduction

Posttraumatic stress disorder (PTSD) is common, typically chronic, and associated with high rates of psychiatric comorbidity. In about 30–50% of patients with PTSD, marked symptoms persist after treatment (1) and severely impact quality of life (2, 3). However, a sizable minority of veterans who initially experience prominent symptoms of PTSD eventually experience remission (4, 5). A reassessment after 40 years of veterans who participated in the National Vietnam Veterans Readjustment revealed that while PTSD symptoms worsened for theater veterans with PTSD as a whole, there were 7.6% who showed significant clinical improvement (4). A similar large-scale longitudinal re-examination of the Vietnam era twins after 20 years identified 17% of theater veterans developed PTSD early and experienced early symptom remission, and 7.4% who developed PTSD early and experienced late symptom remission (5). These results clearly highlight relapse and remission in PTSD (6, 7). Some recent studies have investigated the neural changes associated with the remission of PTSD in response to specific therapeutic interventions (8–10). However, our understanding of the associated neurostructural and network correlates of remission is still limited.

A wealth of research demonstrates that PTSD is associated with an array of functional and anatomical changes at specific anatomical loci in the brain, including amygdala, hippocampus, insula, ventral/dorsal medial/lateral prefrontal cortex, and anterior/posterior cingulate cortex (11–14). However, the human brain is organized into complex networks (15) supported by long range connections (16) that may be modified by exposure to traumatic events, precipitate alterations in network topology, and ultimately in behavior/symptoms (17, 18). Changes in network topology can be conveniently quantified by graph theoretical measures to understand changes in associated behavior and neuropsychiatric symptoms (i.e., dysfunctional behaviors) (19–21). Network architecture can be inferred from various neuroimaging methods including functional resting or task-based functional magnetic resonance imaging (fMRI), fiber connectivity from diffusion tensor imaging of white-matter tracts, and structural brain network derived from between-subject regional correlation of cortical thickness (22). Among them, cortical morphometric network analyses (23) are based on inferences about structural covariance between pairs of cortical regions that covary with respect to cortical thickness or subcortical regions that covary with respect to volume (24). The dependence between brain areas is postulated to derive from structural or functional associations between these regions (22, 24–26). The brain structural network method is relatively immune to a range of noisy components that accompany task-based and resting state fMRI, and purportedly reflects a highly choreographed developmental process of neuronal growth and migration throughout the cortical mantle (24, 27).

Mueller et al. (28) showed PTSD-associated alterations of structural brain networks, demonstrating an enhanced role of the left insula and right orbitofrontal cortices (betweenness centrality) and a diminished role of left orbitofrontal and anterior cingulate cortices (degree centrality) within the network. Centrality is a mathematical measure from graph theory that characterizes the importance of a particular region within a network of connections (edges) between brain regions (nodes). We investigated the network characteristics of remitted PTSD (lifetime but not current diagnosis) by comparing the centralities of structural networks to those in patients with current PTSD and trauma-exposed control subjects without lifetime PTSD. Our goal was to identify structural network characteristics associated with PTSD and its remission. Such knowledge may help identify potential targets for interventions to ameliorate chronic PTSD by facilitating remission. The disorder is characterized by symptoms and behaviors that produce cognitive impairment particularly with attention and memory (29); difficulty with regulating emotions, particularly in response to threat and fear (30); avoidance, often in the form of social anxiety (31) and hypervigilance, among others (11). Thus, we hypothesized between-group differences of network centrality in cortical regions associated with cognitive function, emotion regulation, fear processing, social cognition, and inhibitory control, by focusing on the prefrontal cortex including anterior cingulate cortex, specifically subcallosal gyrus (32, 33), frontal pole, and superior frontal areas (34, 35) that have been associated with PTSD.

## Materials and Methods

### Participants

Participants (*n* = 317) recruited from a repository (Mid-Atlantic MIRECC Post-Deployment Mental Health Repository, Durham, NC, USA) (36, 37) of Iraq and Afghanistan era military service members underwent structural MRI scans. Participants were screened for inclusion/exclusion criteria based on information available in the repository. Important exclusions included major axis I diagnosis (other than major depressive disorder or PTSD), contraindication to MRI, traumatic brain injury, substance dependence, neurological disorders, and age over 65 years. This study was carried out in accordance with the recommendations of the Institutional Review Boards at Duke University and the Durham VA Medical Center with written informed consent from all subjects. All subjects gave written informed consent in accordance with the Declaration of Helsinki. The protocol was approved by the Institutional Review Boards at Duke University and the Durham VA Medical Center. Participants previously completed questionnaires upon entering the repository that were available for this study to assess traumatic life events [Traumatic Life Events Questionnaire (TLEQ) (38)], combat exposure [Combat Exposure Scale (CES) (39)], depressive symptoms [Beck Depression Inventory-II (BDI-II) (40)], and serotonergic antidepressant medication use (Med_5HT). Diagnosis of PTSD was performed with the Clinician-Administered PTSD Scale to determine three groups (1) current PTSD who met diagnostic criteria based on symptoms experienced in the past month (CURR-PTSD; *n* = 101), (2) lifetime PTSD but no current PTSD for participants who met diagnostic criteria before the last month but not since (REMIT-PTSD; *n* = 35), and (3) those without PTSD who never met diagnostic criteria (CONTROL; *n* = 181). Comorbid psychiatric diagnoses were ascertained upon entering the repository with the Structured Clinical Interview for DSM-IV (SCID). Childhood trauma (child-trauma) was coded from the number of trauma categories experienced before age 18 (e.g., physical abuse, sexual abuse, and serious accident) as reported in the TLEQ (41).

### MRI Acquisition and Analyses

All images were acquired on 3-T scanners equipped with an 8-channel headcoil. The majority (90.6%) of images was acquired on two GE scanners using high-resolution T1-weighted whole-brain axial images with 1-mm isotropic voxels with array spatial sensitivity encoding technique and fast spoiled gradient recall (3D-FSPGR). Image parameters were optimized for contrast between white matter, gray matter, and CSF on the (i) GE Discovery MR750 (*n* = 156, including 79 CONTROL, 21 REMIT-PTSD, and 56 CURR-PTSD) (TR/TE/flip angle = 7.484 ms/2.984 ms/12°, FOV = 256 mm, 1 mm slice thickness, 166 slices, 256 × 256 matrix, 1 excitation) and (ii) GE Signa EXCITE (*n* = 132, including 80 CONTROL, 11 REMIT-PTSD, and 41 CURR-PTSD) (TR/TE/flip angle = 8.208 ms/3.22 ms/12°, FOV = 256 mm, 1-mm slice thickness, 166 slices, 256 × 256 matrix, 1 excitation). The remaining images (9.4%) were collected on a Philips Ingenia scanner (*n* = 29, including 22 CONTROL, 3 REMIT-PTSD, and 4 CURR-PTSD) using higher in-plane resolution 0.9375 mm × 0.9375 mm × 1.0 mm 3D turbo field echo pulse sequence with contrast enhancement and SENSE (TR/TE/flip angle = 8.148 ms/3.728 ms/8°, FOV = 240 mm, 1-mm slice thickness, 170 slices, 256 × 256 matrix, 1 excitation). Chi-square test showed that the group distribution is independent of scanner (χ^2^ = 8.725, df = 4, *p* = 0.068). All T1 images were visually inspected to assure sufficient quality for automated segmentation and labeling, which were performed using the FreeSurfer image analysis suite (version 5.3.0; http://surfer.nmr.mgh.harvard.edu/) and its library tool *recon-all*. Details of FreeSurfer parcellations have been previously described (12, 42–45). Thickness measures were calculated for 148 cortical regions (74 per hemisphere) using the aparc.a2009s template (44) through the FreeSurfer software.

### Network Analyses

We generated interregional partial correlation matrices for each participant group by calculating partial correlation coefficients for all regional pairings of cortical thickness across group members. The partial correlation between two regions represents their relationship after partialing out the effects of potential influences of age, sex, IQ, BDI-II, TLEQ, CES, child-trauma, and Med_5HT. These confounding factors have previously been associated with brain structural volumetry (46, 47). A threshold was imposed on the partial correlation matrices to create a binary graph with connections (edges) between regions (19, 48). Using the same threshold for group comparisons may give the results that reflect not only topological differences but also connectivity strength differences. We were interested in topological differences only and thus adopted group-specific thresholds to ensure that the graphs of all groups had an equal number of edges or wiring cost defined as the number of edges present divided by maximum possible number of edges. This method has been successfully utilized in the recent published works on maltreatment (48) and PTSD (28). We calculated the minimum wiring cost required to produce a fully connected network for each group and chose the largest minimum wiring cost (i.e., 0.4987, which was from the CONT group; minimum wiring costs were 0.0868 for REMIT-PTSD and 0.2075 for CURR-PTSD) across groups to derive the corresponding threshold for each group. This method ensured that all nodes were in the network while minimizing the number of redundant paths. We only kept the positive suprathreshold partial correlations in the networks due to the observation that only positive thickness correlations were mediated by direct fiber pathways (24). The network analyses were conducted using in-house Matlab (R2016b) scripts running on an iMac computer (macOS Sierra, version 10.12.6) (49).

### Centrality Measures

A large array of network topology measures can be calculated for a given network and some provide very similar information to other measures. For ease of comparison to previous studies, we analyzed four types of centrality using Brain Connectivity Toolbox [BCT (50)]: (1) degree centrality—number of connections that a node has, (2) betweenness centrality—frequency with which a node falls between pairs of other nodes when traveling along their shortest interconnecting path, (3) closeness centrality—normalized number of steps required to access every other node from a given node in a network (adapted from the distance function in BCT), and (4) eigenvector centrality—a spectral centrality measure based on the idea that the importance of a node is recursively related to the importance of the nodes associated with it. Graph theory postulates that nodes with high centrality play an important role in communication and information transfer within a network (20, 28, 48). Naturally, the various centrality constructs are sometimes correlated but still reflect different aspects in nodal roles of a brain networks.

### Statistics

The variance in the groups’ measures was determined to be equal despite the disparate sample sizes of the three groups (51). We detected four cortical regions showing unequal variances (left lateral sulcus, right frontal pole, right anterior transverse temporal gyrus, and right temporal pole) through two-sample *F* tests for equal variances (at the 1% significance level) and excluded them from further analysis. Furthermore, we tested the reliability of the centrality measures with the Jackknife resampling method (52) to calculate the 99% confidence interval (CI). The Jackknife resampling method has been successfully utilized in analyzing the network derived from partial correlations (48). Finally, to assess between-group differences, we employed permutation testing to compute the probability that the difference in centrality measures between two groups occurred by chance. The permutation testing was based on 10,000 network comparisons derived by randomly permuting the group label of subjects (20, 53). To control for type 1 error, we followed the procedure by Teicher et al. (48) and employed a more conservative threshold that deems a node differs in centrality between groups only if the permutation-derived *p*-values are ≤0.05 for at least three of four centrality measures, which very conservatively reduces the odds of chance occurrence to ≤0.000125 (0.05 × 0.05 × 0.05). The more conservative threshold was used because we were comparing among three groups while Teicher et al. (48) only compared between two groups. For *a priori* cortical regions, the nodal between-group differences were considered significant when the permutation-derived *p*-values are ≤0.05 for at least two of four centrality measures. The statistical analyses were conducted using in-house Matlab scripts. The code for permutation testing was modified from the GRETNA toolbox (54).

## Results

### Demographic and Clinical Characteristics

Participants’ age, sex, and other demographic and clinical information are summarized in Table 1. The three groups did not significantly differ with respect to age and gender.

**Table 1:** Demographic information.

Test	Mean (SD)^a^			*t*-Statistic (*p*-value)^b^		
	REMIT-PTSD (*n* = 35)	CURR-PTSD (*n* = 101)	CONTROL (*n* = 181)	REMIT-PTSD versus CONTROL	REMIT-PTSD versus CURR-PTSD	CURR-PTSD versus CONTROL
Age	40.9 (10.7)	40.3 (10.0)	39.4 (9.9)	0.829 (0.408)	0.299 (0.765)	0.756 (0.450)
Sex	28 (7)	87 (14)	145 (36)	0.000 (0.988)	0.750 (0.386)	−1.615 (0.204)
IQ	99.6 (11.6)	95.8 (12.3)	101.8 (9.5)	−1.174 (0.242)	1.500 (0.136)	−4.440 (0.001)
BDI-II	10.4 (7.8)	22.2 (12.1)	5.1 (7.8)	3.648 (<0.001)	−5.336 (<0.001)	14.386 (<0.001)
TLEQ	20.5 (11.5)	23.7 (14.0)	12.2 (10.0)	3.999 (<0.001)	−1.138 (0.257)	7.254 (<0.001)
CES	11.1 (11.1)	17.1 (10.6)	6.5 (8.4)	2.572 (0.011)	−2.634 (0.010)	8.412 (<0.001)
Child-trauma	1.0 (1.0)	0.7 (1.0)	0.4 (0.8)	3.168 (0.002)	1.161 (0.248)	2.371 (0.019)
Med_5HT	5 (29)	51 (49)	4 (172)	−10.738 (0.001)	13.740 (<0.001)	−94.888 (<0.001)
AUDIT	4.6 (3.5)	4.2 (5.4)	2.8 (3.4)	2.618 (0.010)	0.362 (0.718)	2.414 (0.017)
CAPS_curr	19.4 (13.9)	68.2 (22.4)	7.0 (11.1)	5.451 (<0.001)	−11.437 (<0.001)	29.119 (<0.001)
CAPS_life	63.5 (21.0)	83.5 (29.6)	14.7 (15.8)	14.885 (<0.001)	−3.462 (0.001)	24.160 (<0.001)
DAST	0.7 (1.5)	1.1 (2.5)	0.4 (0.8)	1.404 (0.162)	−0.923 (0.357)	3.295 (0.001)
DTS	26.3 (24.5)	68.6 (33.7)	9.8 (18.7)	4.145 (<0.001)	−6.324 (<0.001)	17.023 (<0.001)

^a^Values outside/inside brackets are number of either males/females for “sex” or yes/no for “Med_5HT.”

^b^Statistical values are from chi-square tests for “sex” and “med_5HT.”

*CONTROL, control group with trauma exposure; CURR-PTSD, current PTSD group; REMIT-PTSD, lifetime but no current PTSD group; IQ, intelligence quotient; BDI-II; Beck Depression Inventory-II; TLEQ, trauma life events questionnaire; CES, Combat Exposure Scale; Child-trauma, categories of trauma exposure as child/adolescent; Med_5HT, serotonergic medication; AUDIT, Alcohol Use Disorders Identification Test; CAPS_curr, Clinician-Administered PTSD Scale reflecting symptoms in the last 30 days; CAPS_life, Clinician-Administered PTSD Scale reflecting symptoms in the worst 30-day period of subject’s life; DAST, Drug Abuse Screening Test; DTS, Davidson Trauma Scale; PTSD; posttraumatic stress disorder; TLEQ; Traumatic Life Events Questionnaire*.

### Centrality Measures

**Figure 1:**
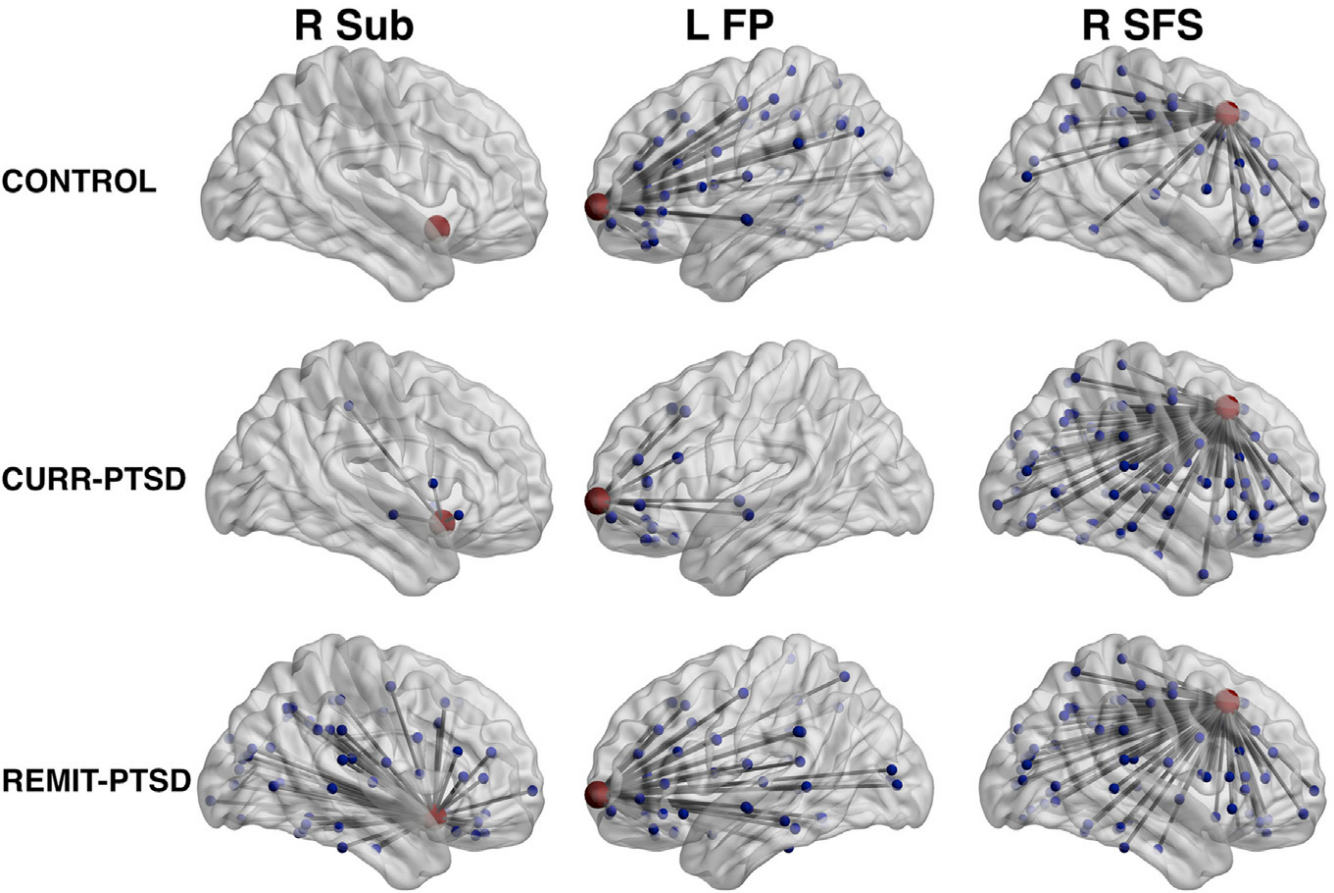
Between-group differences of centrality measures. Left column, REMIT-PTSD (lifetime but no current PTSD group) group showed larger centrality in right subcallosal gyrus (R Sub) than both CURR-PTSD (current PTSD group) and CONTROL (control group with trauma exposure) groups. Middle column, both REMIT-PTSD and CONTROL groups showed larger centrality in left frontal pole (L FP) than CURR-PTSD group. Right column, both REMIT-PTSD and CURR-PTSD groups showed larger centrality in right superior frontal sulcus (R SFS) than CONTROL group. For observation purpose, the regions of interest (red), their connected nodes (blue), and the connections (gray) were shown. Node size was scaled by degree centrality.

The centrality measures of all nodes were within their corresponding 99% CI, supporting the reliability of the analyses. Significant between-group differences of nodal centrality were identified in several cortical regions. Specifically, centrality between-group differences were detected in anterior cingulate cortex, frontal pole and superior frontal areas (see Figure 1).

### REMIT-PTSD Versus CONTROL

As shown in Table 2, REMIT-PTSD patients (versus CONTROL) showed smaller centrality in left paracentral lobule and sulcus and bilateral precentral gyrus. They also showed larger centrality in right subcallosal gyrus, vertical ramus of the anterior segment of the lateral sulcus, superior segment of the circular sulcus of the insula, superior frontal sulcus, and anterior occipital sulcus.

**Table 2:** Centrality between-group comparisons: REMIT-PTSD (lifetime but no current PTSD group) versus CONTROL (control group with trauma exposure).

No.	Area	Degree	Betweenness	Closeness	Eigenvector
**REMIT-PTSD < CONTROL**					
3	L Paracentral lobule and sulcus	41/91	14.0/63.0	0.639/0.803	0.046/0.099
29	L Precentral gyrus	64/117	31.5/194.8	0.718/0.891	0.074/0.119
29	R Precentral gyrus	67/112	60.8/134.7	0.728/0.874	0.073/0.115

**REMIT-PTSD > CONTROL**					
32	R Subcallosal gyrus*	67/0	53.9/0.0	0.728/0.000	0.075/0.000
40	R Vertical ramus of the anterior segment of the lateral sulcus	88/7	111.7/0.0	0.799/0.509	0.095/0.008
49	R Superior segment of the circular sulcus of the insula	120/108	264.4/246.4	0.908/0.861	0.124/0.105
54	R Superior frontal sulcus*	101/58	126.6/19.8	0.844/0.690	0.111/0.063
59	R Anterior occipital sulcus	110/70	210.7/42.6	0.874/0.731	0.116/0.076

At least three of four (two of four in *a priori* areas marked with *) centrality measures showed significant between-group differences in these areas.

*“No.” is the label of cortical area in the aparc.a2009s template (44)*.

*L, left; R, right*.

### REMIT-PTSD Versus CURR-PTSD

As shown in Table 3, REMIT-PTSD (versus CURR-PTSD) patients showed smaller centrality in left precentral gyrus, right precuneus, right middle temporal gyrus, and right inferior frontal sulcus. They also showed larger centrality in left transverse frontopolar gyri and sulci, left posterior-dorsal part of the cingulate gyrus, right lateral occipito-temporal gyrus, and right subcallosal area.

**Table 3:** Centrality between-group comparisons: REMIT-PTSD (lifetime but no current PTSD group) versus CURR-PTSD (current PTSD group).

No.	Area	Degree	Betweenness	Closeness	Eigenvector
**REMIT-PTSD < CURR-PTSD**					
29	L Precentral gyrus	64/105	31.5/108.8	0.718/0.857	0.074/0.112
30	R Precuneus	80/119	59.5/202.1	0.772/0.905	0.090/0.121
38	R Middle temporal gyrus	71/112	76.0/137.9	0.741/0.881	0.074/0.118
52	R Inferior frontal sulcus	66/114	41.0/159.0	0.724/0.888	0.075/0.118

**REMIT-PTSD > CURR-PTSD**					
5	L Transverse frontopolar gyri and sulci	65/18	46.0/1.2	0.721/0.543	0.071/0.011
9	L Posterior-dorsal part of the cingulate gyrus	94/45	154.5/18.5	0.820/0.653	0.097/0.044
21	R Lateral occipito-temporal gyrus	116/83	205.2/97.3	0.895/0.782	0.124/0.081
32	R Subcallosal area*	67/8	53.9/4.0	0.728/0.518	0.075/0.006

At least three of four (two of four in *a priori* areas marked with *) centrality measures showed significant between-group differences in these areas.

*“No.” is the label of cortical area in the aparc.a2009s template (44)*.

*L, left; R, right*.

### CURR-PTSD Versus CONTROL

As shown in Table 4, CURR-PTSD patients (versus CONTROL) showed smaller centrality in left transverse frontopolar gyri and sulci, left posterior-dorsal part of the cingulate gyrus, left postcentral gyrus, left inferior segment of the circular sulcus of the insula, and right anterior transverse collateral sulcus. They also showed larger centrality in left sulcus intermedius primus (of Jensen), right precuneus, right inferior/middle/superior frontal sulcus, right lateral orbital sulcus, and right inferior part of the precentral sulcus.

**Table 4:** Centrality between-group comparisons: CURR-PTSD (current PTSD group) versus CONTROL (control group with trauma exposure).

No.	Area	Degree	Betweenness	Closeness	Eigenvector
**CURR-PTSD < CONTROL**					
5	L Transverse frontopolar gyri and sulci	18/68	1.2/56.6	0.543/0.724	0.011/0.073
9	L Posterior-dorsal part of the cingulate gyrus	45/104	18.5/241.0	0.653/0.847	0.044/0.104
28	L Postcentral gyrus	57/92	7.7/52.4	0.686/0.806	0.063/0.101
48	L Inferior segment of the circular sulcus of the insula	51/105	107.4/151.0	0.673/0.850	0.042/0.105
50	R Anterior transverse collateral sulcus	19/62	1.0/26.1	0.557/0.704	0.023/0.068

**CURR-PTSD > CONTROL**					
55	L Sulcus intermedius primus (of Jensen)	47/11	5.9/0.0	0.653/0.514	0.052/0.012
30	R Precuneus	119/91	202.1/48.0	0.905/0.803	0.121/0.100
52	R Inferior frontal sulcus	114/90	159.0/101.0	0.888/0.799	0.118/0.095
53	R Middle frontal sulcus	99/31	150.4/13.2	0.837/0.598	0.100/0.029
54	R Superior frontal sulcus*	115/58	339.8/19.8	0.891/0.690	0.114/0.063
62	R Lateral orbital sulcus	62/14	50.7/1.2	0.711/0.536	0.068/0.014
68	R Inferior part of the precentral sulcus	112/81	183.5/75.3	0.881/0.769	0.117/0.089

At least three of four (two of four in *a priori* areas marked with *) centrality measures showed significant between-group differences in these areas.

*“No.” is the number of cortical area in the aparc.a2009s template (44)*.

*L, left; R, right*.

## Discussion

We characterized neuroanatomical networks defined by cortical thickness correlations for comparison between groups with current PTSD, remitted PTSD, and trauma-exposed controls. Consistent with our *a priori* hypotheses, significant differences between REMIT-PTSD and CURR-PTSD as well as CONTROL participants were detected in the right subcallosal gyrus, frontal pole (left transverse frontopolar gyri and sulci) and superior frontal sulcus. Network connectivity of the frontal pole in REMIT-PTSD appeared to assume a connectivity profile that is consistent with trauma-exposed controls, whereas the CURR-PTSD group had altered frontopolar network connectivity. On the other hand, the REMIT-PTSD group demonstrated brain network architecture at the superior frontal sulcus that was consistent with CURR-PTSD but clearly altered compared with CONTROL despite the lack of PTSD symptoms. A precise neurobiological interpretation of the between-group differences in the covariance of cortical thickness between regions is yet unclear. It is possible that the correlation strength increases between regions that are concurrently affected by disorder (or recovery) processes, which are perhaps due to loss of (or new) input from directly affected regions, and the correlation strength decreases between affected and unaffected regions (28). Our result is consistent with previous findings that frontopolar cortical thinning is associated with increased PTSD symptom severity (35) but to the best of our knowledge this is the first published report to directly compare remitted PTSD to current PTSD, and trauma-exposed controls.

The REMIT-PTSD and CONTROL groups showed larger centrality in the left frontal pole than the CURR-PTSD group, thereby implicating this area in current PTSD symptoms. We posited that lower frontal pole centrality in CURR-PTSD is associated with non-synchronized cortical thickness changes with regions involving cognitive, social, emotional, and most notably self-referential processing (55). This idea is consistent with previous findings that PTSD patients showed altered functional connectivity within brain structures associated with default model network (DMN), which plays a key role in self-referential processing and social cognition (56). The DMN consists of several areas including the anterior medial cortices. Interestingly, REMIT-PTSD and CONTROL groups did not differ in left frontopolar centrality. Clinically, PTSD associates with disturbances in self-referential processing whereby trauma undermines a sense of adaptive self-agency (57). Other closely related cognitive constructs of self-reference that are negatively impacted by PTSD include shame, guilt, self-blame, and a fragmented self-image (58–60). Key neural correlates of these self-referential cognitions are situated in the frontal pole (12, 55). Accordingly, PTSD exhibits functional differences in the frontopolar area for behavioral challenge tasks (11) and during the resting state (61). It is yet unclear whether the higher centrality in REMIT-PTSD (versus CURR-PTSD) patients reflects a recovery of network topology back to a configuration that was present before onset of PTSD, or a compensatory re-organization of network topology to comparable centrality at the frontal pole. These interpretations will require testing in future studies on the role of the frontal pole in remitted and current PTSD and could be explored as a potential target for therapeutic intervention. A recent study using transcranial direct current stimulation (tDCS) has already targeted ventromedial prefrontal cortex for the treatment of PTSD (62). Future clinical intervention on PTSD may utilize interventions including tDCS and deep brain stimulation targeting frontal pole.

The REMIT-PTSD group showed larger centrality in right subcallosal area than CURR-PTSD and CONTROL groups, suggesting the role of subcallosal area in representing the specific status of remitted PTSD patients. The subcallosal area is located in subgenual anterior cingulate cortex and is strongly associated with emotion processing (63). Reduced volume in this area was reported in combat-related PTSD patients compared with trauma-exposed controls (32) and cortical thinning in PTSD remitters compared with non-remitters following treatment with prolonged exposure and trauma-exposed healthy volunteers (9). The subcallosal area also connects with several cortical and subcortical structures including prefrontal cortex, cingulate cortex, amygdala, and hippocampus (64), which have been implicated in PTSD based on anatomical and functional changes (11, 12, 14). However, our findings demonstrated that concurrent changes in cortical thickness in the subcallosal area and its structurally correlated regions were present only in the REMIT-PTSD group, but not in CURR-PTSD (compared with CONTROL). It is possible that a compensatory re-organization of network topology with larger centrality at right subcallosal area contributes to the resilience after experiencing PTSD symptoms by promoting symptom remission. However, we cannot exclude the possibility that some patients have special network topology with greater centrality in subcallosal area that helps with remission from PTSD symptoms. Indeed, US Special Forces who demonstrated resilience in the face of severe trauma have enhanced subcallosal activity during expectation of reward (65).

The REMIT-PTSD and CURR-PTSD groups showed larger centrality in right superior frontal sulcus than the CONTROL group, possibly implicating an enduring marker of prior PTSD even when symptoms remit. Our findings are consistent with previous reports that reduced cortical thickness in the superior frontal area is associated with PTSD symptom severity (34, 35). The superior frontal regions (covering both superior frontal sulcus and gyrus) have been associated with high-level cognitive functions such as working memory and inhibition control (66, 67), which contribute to downregulating the personal relevance of the negative events (68). Structural and functional connectivity methods show that the superior frontal area consists of subregions that connect with nodes of the cognitive executive network (including middle and inferior frontal gyrus), default mode network (including anterior and middle cingulate cortex), and motor control network (including precentral gyrus, caudate, thalamus, and frontal operculum) (69). Thus, our results suggest that a high centrality in the right superior frontal sulcus represents lifetime PTSD-related neural changes in brain areas typically associated with executive, motor, and self-processing. Given that PTSD is a chronic relapsing and remitting disorder (4, 70), it is possible that some neural changes persist following symptom remission and may serve as a biomarker of possible future relapse. It is possible, although unlikely, that these network connectivity changes are precipitated by trauma in certain individuals given the absence of this neural configuration in the trauma-exposed group. However, given that childhood trauma that is associated with lasting brain changes (71), we cannot exclude the possible influence of higher childhood trauma scores in the CURR-PTSD and REMIT-PTSD patients.

Our study provided comparable results to previous structural network analyses on individuals with PTSD. We found larger centrality in right lateral orbital sulcus in individuals with CURR-PTSD than in CONTROL subjects, which is consistent with Mueller et al. (28) who report larger betweenness centrality in the right orbitofrontal area of veterans with PTSD obtained from either a whole-brain network analysis or a network analysis restricted to prefrontal-limbic areas. These convergent findings suggest that larger centrality in right orbitofrontal area is associated with PTSD symptoms. Previous studies have detected reduced gray matter volume/concentration in orbitofrontal cortex in patients with either PTSD (72) or depression (73). In line with these results, the larger centrality in right orbitofrontal area in CURR-PTSD patients may reflect reduced gray matter in both right orbitofrontal area and its connected regions. No significant differences of centrality in this area were detected between REMIT-PTSD group and the other groups.

Various factors influence remission, including the trauma type, chronicity of trauma exposure, comorbid substance use, and others (74). In a large meta-analysis of 42 studies with a total of 81,642 participants, 44% of individuals with PTSD experienced spontaneous remission (without specific treatment) at a mean follow-up duration of 40 months (75). Given that we did not specifically assess the antecedents of PTSD remission, it is likely that the present sample was comprised of a mix of patients who experienced spontaneous remission, pharmacotherapeutic remission, psychotherapeutic remission, and some combination thereof. While spontaneous remission is perhaps of particular interest since it represents a form of resilience to PTSD-onset following trauma exposure, the ability to remit in response to treatment is equally interesting because it represents its own form of resilience when contrasted with patients who suffer chronic persistent PTSD and are refractory to treatment. Thus, our REMIT-PTSD sample represents a resilient sample, albeit heterogeneous, composed of a mix of individuals who recovered spontaneously and others who responded to treatment. Future research will be required to dissect the cortical network changes that are unique to each of the various subtypes of remitted patients.

The CURR-PTSD patients showed more severe depression and PTSD symptoms than REMIT-PTSD individuals, suggesting that the differences between CURR-PTSD and REMIT-PTSD patients might be confounded with the severity of depression and PTSD. However, a recent study showed that high depression severity is accompanied with greater centrality in ventral medial prefrontal cortex and posterior cingulate cortex, and smaller centrality in temporal areas and middle/inferior frontal areas (76), which are largely contrary to our findings of the comparison between CURR-PTSD and REMIT-PTSD patients. Therefore, depression severity should not be a confounding factor when explaining our findings. On the other hand, veterans with PTSD were found to be associated with decreased degree centrality in medial orbital frontal areas and rostral cingulate cortex (28), consistent with our contrast between CURR-PTSD and REMIT-PTSD patients. We thus cannot reject the hypothesis that patients with less severe PTSD symptoms are more amenable to remission. Future studies on CURR-PTSD and REMIT-PTSD patients who are matched for lifetime PTSD scores should further address this issue.

### Limitations

There are several limitations in this study. First, our analysis was based on the large-scale covariance of cortical thickness. Some subcortical regions important to PTSD, such as amygdala and hippocampus (14, 77), were not considered. Recent studies have included both cortical thickness and subcortical volumes in structural network analyses (28) although the reliability of this method still needs validation. Future studies should delineate the role of subcortical structures in the brain network of REMIT-PTSD patients. Second, the role of individual differences on network characteristics is unclear, given that a network is defined at the group level. A new approach for investigating cortical thickness networks is needed to investigate the relationship between cortical thickness-based network attributes and individual characteristics (e.g., age and gender). An single-subject gray matter graph method has been developed in recent years (78, 79) and should be utilized in future studies of PTSD and remission. Furthermore, other connectivity analyses methods such as resting state fMRI, based on interregional relationship across measures within each subject, may complement our understanding of the effect of individual difference in network topography. Third, our study utilized a cross-sectional design, which limits inferences about the causal relationships between PTSD remission and cortical thickness network. Future longitudinal studies should fill this gap and explore the relationship between brain network characteristics and treatment outcomes. Longitudinal studies (4, 5) assessing PTSD at two time points to assess the course of illness would be more reliable and robust than a cross-sectional approach because it does not rely on patients’ memories of prior symptoms from the distant past. Last but not least, it is a challenge to estimate the sample size appropriate for a structural covariance network analysis. Conventional power analysis makes comparisons of the group means and variances of the same measures from individuals. By contrast, in our study, no centrality measures can be calculated at the individual subject level that can be pooled for group means and variances. Moreover, connection topology at each node determines the centrality, which means that each graph (group) has 148 relevant centrality measures to consider in any power or sample size calculation. The centrality at each node is not available as a mean and pooled variance that is derived from all subjects in that group. Furthermore, the large number of nodes at which centrality is compared between groups is a separate but related concern about adjusting power for multiple comparison testing. At this stage the field of connectomics, which is based on graph theoretical measures is yet grappling to develop appropriate corrections for multiple comparison testing that may be deployed on graph theoretical measures, and is even further from reaching consensus on best practices (80).

### Conclusion

Our methods and results advance our understanding of the network configuration defined by structural relationships within REMIT-PTSD patients and may offer therapeutic targets for PTSD. Cortical thickness networks, specifically centrality of the right subcallosal gyrus, left frontal pole, and right superior frontal sulcus, differ between remitted PTSD, current PTSD diagnosis, and trauma exposure without PTSD. Our findings in REMIT-PTSD show enhanced structural connectivity that may represent a marker of resilience by promoting symptom remission through a recovery of network topology to a premorbid configuration or a compensatory re-organization of network topology with comparable centrality features.

## Mid-Atlantic MIRECC Workgroup

The Mid-Atlantic MIRECC Workgroup includes the following contributors: Jean C. Beckham, Mira Brancu, Patrick S. Calhoun, Eric Dedert, Eric B. Elbogen, Kimberly T. Green, Robin A. Hurley, Jason D. Kilts, Nathan Kimbrel, Angela Kirby, Christine E. Marx, Gregory McCarthy, Scott D. McDonald, Marinell Miller-Mumford, Scott D. Moore, Rajendra A. Morey, Jennifer C. Naylor, Treven C. Pickett, Jared Rowland, Jennifer J. Runnals, Cindy Swinkels, Steven T. Szabo, Katherine H. Taber, Larry A. Tupler, Elizabeth E. Van Voorhees, H. Ryan Wagner, Richard D. Weiner, Ruth E. Yoash-Gantz.

## Ethics Statement

All subjects gave written informed consent in accordance with the Declaration of Helsinki. The protocol was approved by the Institutional Review Boards at Duke University and the Durham VA Medical Center.

## Author Contributions

All of the authors made substantial contributions to the conception or design of the work; the acquisition, analysis, or interpretation of data for the work; drafting the work or revising it critically for important intellectual content; final approval of the version to be published; and agreement to be accountable for all aspects of the work in ensuring that questions related to the accuracy or integrity of any part of the work are appropriately investigated and resolved.

## Conflict of Interest Statement

The authors declare that the research was conducted in the absence of any commercial or financial relationships that could be construed as a potential conflict of interest.
